# Lost in translation—the use of remote and on-animal sensing for extensive livestock systems

**DOI:** 10.1093/af/vfab049

**Published:** 2021-10-20

**Authors:** David G Masters

**Affiliations:** School of Agriculture and Environment, M085, University of Western Australia, Crawley, WA, Australia

**Keywords:** cattle, grazing, sensors, sheep

**Implications**

- Remote and on-animal technology has been used successfully for intensive animal production where process control is possible. Although grazing livestock systems are different, there is vast potential to use similar technology for industry transformation.
- Despite this, technology adoption has been slow and the transformations predicted 10-15 years ago have not eventuated.
- The current paradigm is not working implying a change is required. It is advocated that a new research model based on requirements for technological packages, designed through a consultative process involving farmers, adoption specialists, engineers and scientists be used to set priorities for future research.

## Introduction

Remote and on-animal sensors underpin the development of Precision Livestock Farming (**PLF**). These technologies have been demonstrated in intensive livestock production resulting in decreased labor costs and in the establishment of animal production as an industrial process (Berckmans, 2017). This level of process control is not appropriate for extensive, large-scale pastoral, and mixed farm grazing. By definition, this is an industry sector where manual inputs must be minimized, the interaction between livestock and the environment is a priority, and management flexibility is essential. Nevertheless, the opportunity to monitor animals remotely has many attractions and it is appropriate to review how research and development over the past 20 yr have progressed toward application.

## Technology for Cropping

The use of geographical positioning systems (GPS), geographical information systems (GIS), remote sensing, and other information technology is now widespread in broadacre cropping. This cropping can be carried out during short but intensive activity periods using large machinery. The technology allows precise applications of fertilizer, seed, and herbicide best suited for specific soils and topography. The machinery does not need to be driven but can be monitored from within an air-conditioned cab or even from a remote location (Mulla, 2013; Tayari et al., 2015). Technological development is progressing rapidly with options such as the use of unmanned aerial vehicles (**UAV**s) for spraying and crop monitoring close to application (Radoglou-Grammatikis et al., 2020). This application and adoption demonstrate that there are appropriate technical skills in the rural sector and a willingness of progressive farmers to adopt change and new technology. Parallel changes have not occurred in the grazing livestock industry suggesting that appropriate technology packages are not available.

## Remote and On-Animal Sensors for Livestock

**Figure 1.**
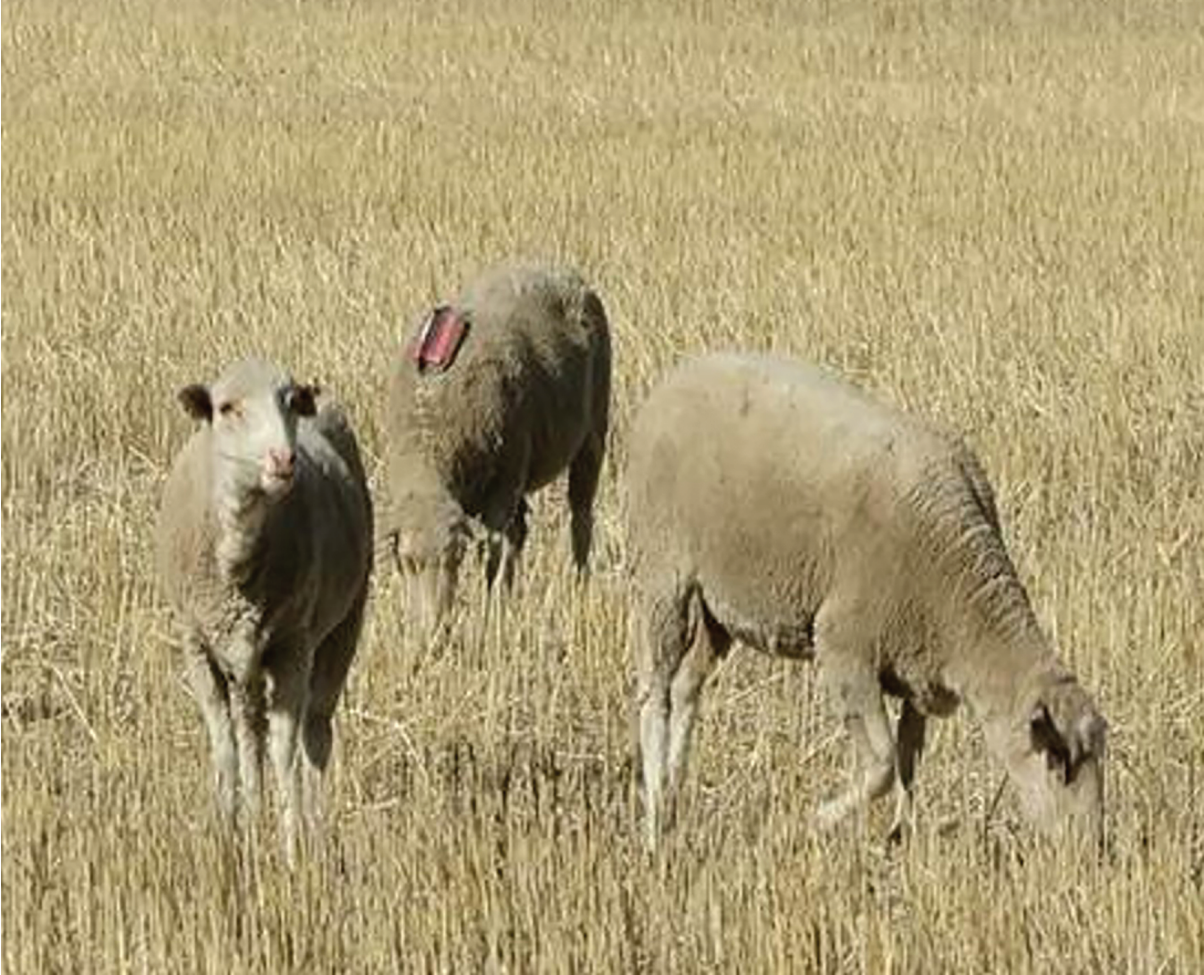
Sheep fitted with a waterproof location and behavior sensor (photo credit Dean Thomas).

The potential for the use of remote and on-animal sensing to improve the production and welfare of grazing livestock and enhance landscape management is transformational. High-value quantitative information will enable a step change in the precision management of pastures and grazing sheep and cattle (Bailey et al., 2021). Opportunities for remote monitoring and management include pasture growth rates and feed on offer from satellite imagery (Hill et al., 2004; Mata et al., 2004), walk over weighing (Brown et al., 2014; González-García et al., 2018), virtual fencing (Umstatter, 2011; Marini et al., 2018), remote drafting for supplement management (Bowen et al., 2009), UAVs for tracking and control (Yaxley et al., 2021), and the use of cameras or sensors for monitoring water points (Bailey et al., 2021). Pasture monitoring provides an indicator of available feed in a constantly changing and complex mosaic of pasture phenologies. Virtual fencing, remote drafting, gate management, and application of UAVs for movement control may support active management not simply monitoring of activity. On or in-animal sensors (Figure 1) can be used to measure a range of different behaviors and characteristics such as distances walked, direction, velocity, acceleration, posture, location, proximity to other animals, body movements (e.g., jaw and bite), body temperature, heart rate, and mounting activity (Fogarty et al., 2018). The collected data have then been related to various indicators of health, production, or welfare such as heat stress and water requirements (Thomas et al., 2008), metabolizable energy intake (Suparwito et al., 2021), parasite infection (Falzon et al., 2013), dog or wolf predation (Manning et al., 2014; Clark et al., 2020), estrus detection (Alhamada et al., 2016), parturition time (Miller et al., 2020), and dam parentage (Brown et al., 2011). There is potential for much more; for example, a recent publication describing the relationship between observed behavior and flystrike is a candidate for an on-animal sensor (Grant et al., 2019), and the relationship between time spent grazing, feed availability, and quality and ground cover (Allden and McDWhittaker, 1970) also has great potential but has not been adequately investigated. In Australia, where one person (with the help of occasional contractors) may manage 5,000 to 10,000 sheep spread across many paddocks and flocks, collection of even a small proportion of the stream of information available from remote sensing through manual measurement and observation would be impossible. Similar conclusions apply to cattle grazing rangelands and extensive pastures. This means that the effective use of remote and on-animal sensors would lead to improvements in efficiency of feed use, environmental management of the farm landscape, welfare, and production and not just to reduced labor costs.

## Adoption and Utilization

Despite the demonstrated capabilities of the technologies, application to facilitate remote PLF has been almost nonexistent. Predictions made in 2012 that PLF would revolutionize the livestock industry in the next 10 yr and that sensors would be deployed routinely around animals to allow farmers to effectively monitor a range of useful parameters (Banhazi et al., 2012) have not eventuated. Why has progress has been so slow? This question has been partly addressed in a review by Bahlo et al. (2019), in which the authors state “applications are mostly based on a single or limited number of indicators or sensors, rather than the desired large number of indicators, which would improve quality of the application.” This is clearly true as 68 of the 71 peer-reviewed articles listed by Fogarty et al. (2018) described the use of either one or two on-animal sensors; moreover, results are usually presented in the context of the individual animals rather than flocks or whole farm. Bahlo et al. (2019) go further and identify the lack of interoperability of various applications as a major challenge. All of this indicates that end users of the technology are not being supplied with solutions. Complex data flows from multiple sources are not useful; farmers will want to turn on a computer or phone at a remote location and be provided with integrated information on feed and water availability, animal weight and condition, and potential health and welfare to support day-to-day management activities.

## Future Research and Development

**Figure 2.**
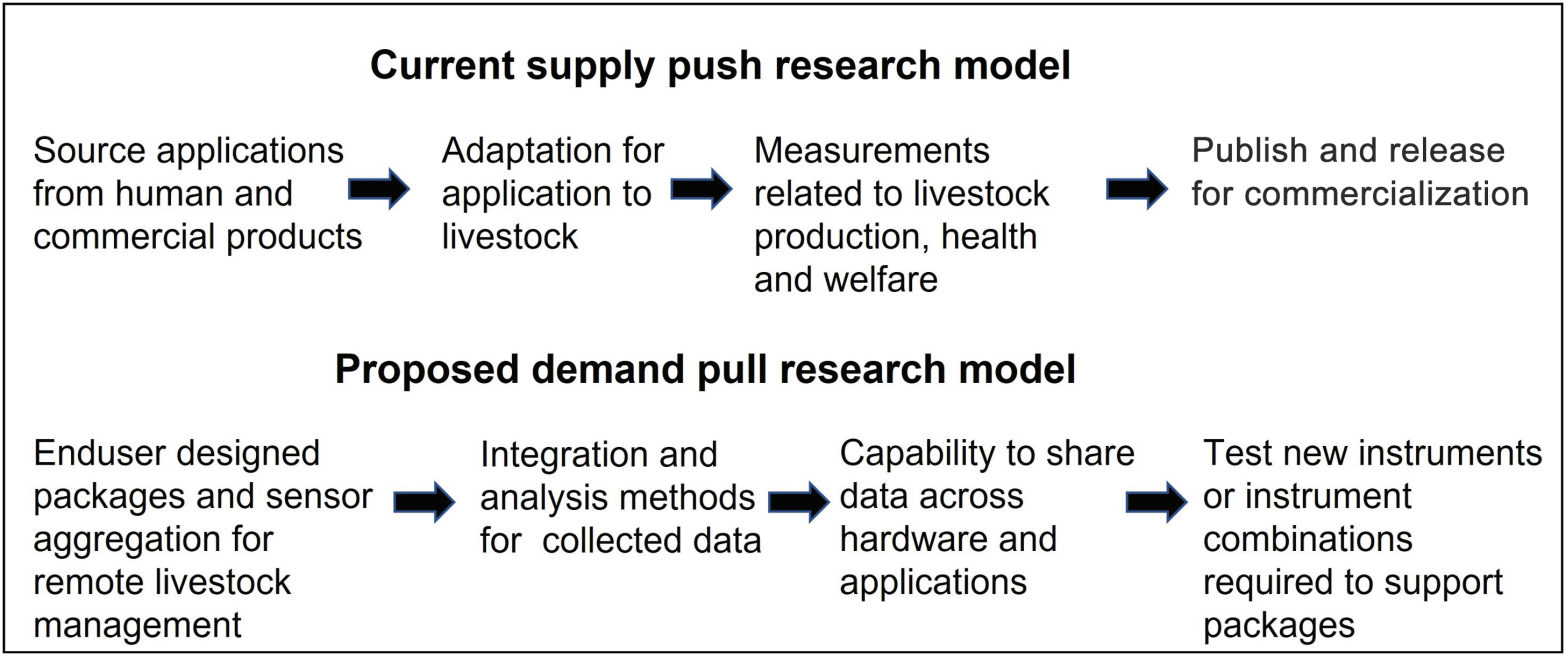
Schematic of the current and proposed approach to the development of technologies for remote livestock management.

It is time to reset the research direction and priorities on the use of technology for grazing systems. There are three clear priorities that can be derived from gaps in published research. The first priority is to establish end user interest and direction. Without enthusiastic support, direction, and commitment by livestock managers, the technology is likely to stall. Only through a well-organized industry consultation process can an end product(s) be designed that will have a chance of adoption. This should include the definition of the questions and solutions that livestock managers consider a priority, reviewed in the context of what technology can deliver, and how it could be packaged. This will also inform the process of sensor aggregation to measure multiple parameters. Very similar comments were made by others 13 yr ago (Wathes et al., 2008) but appear to have been ignored. After consultation, the second priority is integration and intelligent analysis of the collected data to meet design requirements. For example, plentiful pasture accompanied by low intake and poor growth could contribute to the interpretation of behavior change consistent with flystrike, parasites, or nutrient imbalance. This type of conclusion requires the interpretation of multiple data sources. Finally when the product(s) have been designed and a logical framework for data analysis and interpretation completed, the capability to share relevant data across different hardware and applications (interoperability) (Bahlo et al., 2019) will be needed. This process should be demand driven (Figure 2). In reviewing future research and development directions, it is clear that progress will only be made through the formation and support of multidisciplinary teams of engineers, livestock and pasture scientists, adoption experts, and end users.

## About the Authors

**David Masters** is an Adjunct Professor at the University of Western Australia in the School of Agriculture and Environment and an Honorary Fellow with CSIRO. He has a background in nutritional physiology and biochemistry with a specific interest in mineral metabolism. During his 30 yr research career, he has channeled his scientific background into research on applied livestock systems and particularly into managing grazing livestock for positive environmental outcomes. He initiated national programs to reduce the impact of salinity in Australian agriculture through the use of grazing plants and has published on both greenhouse gas from grazing livestock and remote monitoring of movement and behavior. Recent reviews cover topics such as halophytes in grazing systems, physiological changes associated with genetic selection, dystocia in sheep, and the practical implication of mineral deficiencies in grazing livestock.
